# Improving care and reducing costs in oncology

**DOI:** 10.3332/ecancer.2013.ed31

**Published:** 2013-12-03

**Authors:** Giovanni Codacci-Pisanelli

**Affiliations:** University of Rome “la Sapienza”, Department of Medical and Surgical Sciences and Biotechnology, Corso della Repubblica 76, 04100 Latina, Italy

The costs of healthcare are a cause of concern throughout the world. This is the case for Europe, where most of the service is paid for by a state healthcare system, but also in the USA, where private insurance has a more prominent role. Several articles addressing this issue, i.e. the causes of this sharp increase and how to reduce the costs of medicine, have appeared in medical journals in recent months. 

Medical oncology is one of the disciplines where expenses are most obvious, even if doctors do not generally notice the price tag on the drugs they prescribe. Prices in the order of 5000€ per month per patient for a single anticancer drug are not uncommon. I will not discuss here the reasons why pharmaceutical companies charge such prices, as this question may be less important than generally considered since the duration of treatment is often limited to a few months (progression-free survival is often very short) and, in the case of targeted agents, only a selected patient population is (or should be!) involved. 

In a recent paper [1], ASCO underlines five points that are often forgotten when we measure the costs of treatments, updating the recommendations published in 2012 [2]. This list is in some aspects surprising since it includes items that are often overlooked when we think about the price of cancer care: expensive antiemetics, combination chemotherapy, target agents when no target is present. Cautionary use of PET for tumour staging, and follow-up and PSA testing have been reiterated as they were already present in the recommendations issued in 2012. Other items in the 2012 list were to avoid chemotherapy in subjects unlikely to benefit from it and to use granulocyte-stimulating factors only when the risk of febrile neutropenia is not negligible. 

The most important outcome of reading and reflecting on this paper is that it will hopefully encourage oncologists to analyse their clinical habits and to reduce those that are not only expensive, but also of questionable benefit to the patient. Once more it becomes evident that the most important source of expenses is the pen of the doctor [3]. It will be important for doctors to request fewer radiological tests (including ultrasound scans) which will not only save money but will shorten waiting lists – an issue that, in Italy, is one of the most commonly criticised aspects of the healthcare system. 

The next step is to look for further ways to reduce costs, but a tentative shortlist may include: the use of CT scans in terminal patients, requesting whole-abdomen ultrasound scans when we are only interested in the liver, measuring bone density in every woman treated with aromatase inhibitors, prescribing echocardiograms for all breast cancer patients receiving adjuvant anthracyclines with a cumulative dose that is well below the toxic range, using expensive anticancer agents in the third or fourth line of treatment of advanced cancer, using pegylated granulocyte growth factors when not required and prolonging treatment with erythropoiesis-stimulating agents when no response can be expected. 

The price of anticancer drugs, however, cannot be overlooked. Of course the pen of the prescribing oncologist is partly to blame for the overuse of expensive drugs [3] but it has more to do with the regulating authorities. They should require evidence not only of “statistical significance” but also of “clinical relevance” of results obtained with new and super-expensive drugs and even after approval they can still intervene to negotiate favourable prices, as NICE (The National Institute for Health and Care Excellence) have done in the UK [4]. 

Calculating the real value of a test for the patient before prescribing it would be an excellent exercise for every physician, even if many will object that low cost is by definition low quality medicine. In oncology this concern appears irrelevant since it has been shown that when costs were reduced the quality of assistance improved [5],[6]. 

Medical oncologists in the USA seem to be more conscious about costs than we are in Europe, but it is time for us here in Europe to start considering this element since paying more attention to our everyday practice will result in paying less for medical care while maintaining the same quality. 

## List 2013

1. Do not give patients starting a chemotherapy regimen that has a low or moderate risk of causing nausea or vomiting antiemetic drugs intended for use with a regimen that has a high risk of causing nausea or vomiting
2. Do not use combination chemotherapy (multiple drugs) instead of chemotherapy with one drug when treating an individual for metastatic breast cancer unless the patient needs a rapid response to relieve tumor-related symptoms
3. Avoid using positron emission tomography or positron emission tomography–computed tomography scanning as part of routine follow-up care to monitor for cancer recurrence in asymptomatic patients who have finished initial treatment to eliminate the cancer unless there is high-level evidence that such imaging will change the outcome
4. Do not perform prostate-specific antigen testing for prostate cancer screening in men with no symptoms of the disease when they are expected to live fewer than 10 years
5. Do not use a targeted therapy intended for use against a specific genetic aberration unless a patient’s tumor cells have a specific biomarker that predicts an effective response to the targeted therapy.

## List 2012

1. Do not use cancer-directed therapy for patients with solid tumors who have the following characteristics: low performance status (3 or 4), no benefit from prior evidence-based interventions, not eligible for a clinical trial, and with no strong evidence supporting the clinical value of further anticancer treatment
2. Do not perform PET, CT, and radionuclide bone scans in the staging of early prostate cancer at low risk for metastasis
3. Do not perform PET, CT, and radionuclide bone scans in the staging of early breast cancer at low risk for metastasis
4. Do not perform surveillance testing (biomarkers) or imaging (PET, CT, and radionuclide bone scans) for asymptomatic patients who have been treated for breast cancer with curative intent
5. Do not use white cell–stimulating factors for primary prevention of febrile neutropenia for patients with less than 20% risk for this complication.
